# Clinicopathological Analysis of Bronchiolar Adenoma Lined Purely by Mucinous Luminal Cells

**DOI:** 10.1155/2023/5566499

**Published:** 2023-10-31

**Authors:** Guangjie Liao, Xinke Zhang

**Affiliations:** ^1^Department of Pathology, Red Cross Hospital of Yulin, Yulin, China; ^2^Department of Pathology, State Key Laboratory of Oncology in South China, Collaborative Innovation Center of Cancer Medicine, Sun Yat-sen University Cancer Center, Guangzhou, China

## Abstract

Bronchiolar adenoma (BA) is a rare lung tumor that has recently been clearly named, including the previous ciliated muconodular papillary tumor (CMPT) and the so-called nonclassical CMPT. The most prominent histological feature of BA is a double-layer cell structure composed of a continuous basal cell layer and a luminal cell layer. BA lined purely by mucinous luminal cells is very rare, and only one case has been reported in the English literature. This type of BA can easily be misdiagnosed as early mucinous adenocarcinoma. This article analyzes the clinicopathological characteristics of a newly discovered case of BA lined purely by mucinous luminal cells and fully integrated with the literatures.

## 1. Introduction

Bronchiolar adenoma (BA) is a new name for a rare benign tumor of the lung, which was adopted in the fifth edition of the WHO classification of chest tumors in 2021. BA is divided into proximal type (which is similar to proximal bronchiole epithelium with papillary structure, usually composed of mucous cells and ciliated columnar cells) and distal type (which is similar to respiratory bronchiole epithelium with flat luminal surface, without mucus cells and ciliated cells). The most prominent histological feature of BA is the double-layered cell structure composed of continuous basal cell layer and luminal cell layer that is composed of mucous cells, ciliated cells, Clara's cells, and/or type II alveolar epithelial cells in different proportions [1]. Both BA and so-called ciliated muconodular papillary tumor (CMPT) are considered to be rare tumors. To date, a total of 66 cases have been reported in the English literatures [1–20]. In addition, the solitary pulmonary papilloma located in the peripheral lung bronchioles described in the literatures may be one of the lineages of BA [21, 22]. We have recently discovered a case of distal type BA lined purely by mucinous luminal cells, which is extremely rare, and only one case has been reported [23]. This unique case can easily be misdiagnosed as early invasive mucinous adenocarcinoma (IMA). In this article, we analyze this case with its morphological and immunohistochemical characteristics, fully reviewing the literatures. For the convenience of readers, we refer to this rare tumor as BA/CMPT here.

## 2. Data and Methods

### 2.1. General Data

We collected the data of one case of BA/CMPT admitted to the Red Cross Hospital of Yulin City. And we analyzed and summarized its clinical information, surgical findings, imaging examinations, intraoperative frozen pathology, routine pathology, and immunophenotypes. Follow-up information was obtained through telephone consultation with the informed consent of the patient.

### 2.2. Intraoperative Frozen Pathology and Routine Pathology

Fresh specimens were sent to the pathology department for intraoperative frozen pathology. Routine pathological specimens were fixed with 10% neutral formalin; then, the specimens were routinely dehydrated and embedded in paraffin, and 4 *μ*m thick tissue serial sections were performed. After HE staining, the lesions were observed under the microscope.

### 2.3. Immunohistochemistry (IHC)

We adopted EnVision 2-step method in the IHC experiment. All primary antibodies, including TTF-1, NapsinA, CK7, CK5/6, P40, P63, P53, Ki67, SMA, CEA, CK20, MUC1, MUC5AC, BRAF V600E, and EGFR, were all purchased from Shanghai Jiehao Gene Technology Co., Ltd. The determination of positive staining results shall be based on the brown-yellow color. P40, P63, P53, TTF1, and Ki67 were located in the nucleus, and the rest antibodies were located in the cytoplasm. A positive control and a negative control were set separately.

### 2.4. Histochemical Staining

The mucus of tissues was stained with the Alcian Blue-Periodic Acid Schiff (AB-PAS) method. The kit was purchased from Zhuhai Besso Co., Ltd., and the staining steps were performed according to the instructions. Mucus staining was judged to be positive when the cytoplasm appeared light blue.

## 3. Results

### 3.1. General Clinical Data

A 38-year-old female patient was admitted to the hospital because a lung mass was found for 2 days. The lung examination revealed a space-occupying lesion in the middle lobe of the right lung. The patient had no clinical symptoms such as cough, sputum, and fever. The superficial lymph nodes were not palpable. The level of serum markers including CEA, NSE, and AFP showed normal.

### 3.2. Image Data

**Figure 1:**
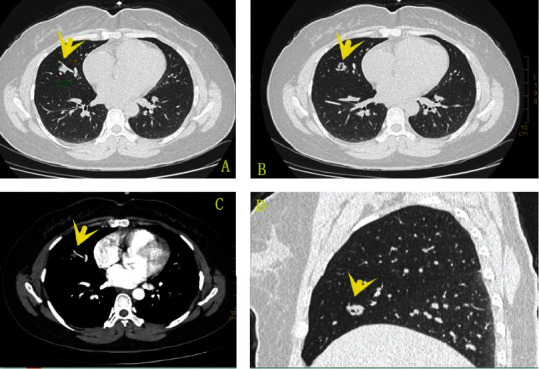
The nodule was located next to the blood vessel and bronchus. The blood vessel and bronchus ran naturally without any involvement (a). There was a small cavity in the nodule, and the inner wall was smooth (b). It was slightly strengthened after enhancement, and the adjacent blood vessel wall was smooth (c). The nodule was lobulated with short burrs in the sagittal plane (d).

The CT examination revealed a nodule in the inner segment in the middle lobe of the right lung. The nodule was about 13 mm × 12 mm × 13 mm in size. The blood vessel and bronchus ran naturally without any involvement (Figure 1(a)). There was a small cavity in the nodule, and the inner wall was smooth (Figure 1(b)). The nodule was slightly strengthened when enhanced by the scan (Figure 1(c)). There were shallow lobes and burrs on the edge (Figure 1(d)). No enlarged lymph nodes were found in the hilar and mediastinum. There is no pleural effusion on both sides of the chest. The radiologists considered the nodule as a lung cancer.

### 3.3. Surgical Findings

The operation of thoracoscopic lobectomy+regional lymph node dissection+closed thoracic drainage was performed. The operator found a grayish-white mucoid mass located in the medial segment of the right middle lobe. Its size was about 1.5 cm × 1.5 cm × 1.5 cm. The section was medium texture, with a small cyst in the middle of the mass. Group 11 lymph nodes of the right lung were swollen.

### 3.4. Pathological Examination and Diagnosis

#### 3.4.1. Intraoperative Frozen Pathological Results

A grayish-white lesion with a size of 1.2 cm × 1.0 cm × 1.0 cm was seen in the lung tissue section. Its boundary was not clear. The lesion was considered as benign lesion in intraoperative frozen pathology. A clear diagnosis needed to be determined after routine paraffin sections.

#### 3.4.2. Routine Pathological Results

**Figure 2:**
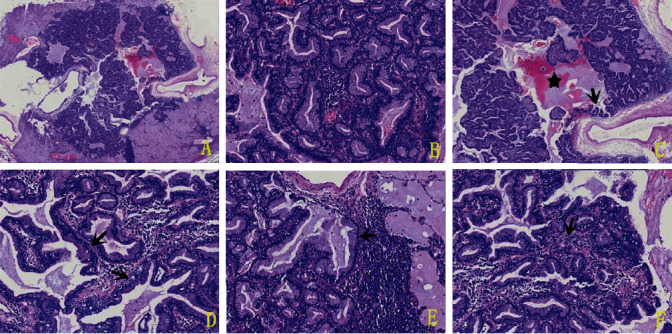
HE staining. Overall view of tumor (a) (40x). Double-layer cell structure, the cavity surface was pure columnar mucinous cells (b) (100x). There was a small amount of papillary (c) (black arrow, 40x), with abundant intracellular and extracellular mucus (c) (black star). Continuous basal cells (d) (black arrow, 200x) can be seen. Basal cells in some areas were not obvious (e) (200x, black arrow). There were abundant stromal cells in some sections (f) (200x, black arrow).

*(1) Histological Changes*. There were two kinds of tumor cells in the lesion, namely, mucous cells and basal cells. Most of them were arranged in an adenoid structure, and a few were papillary structures. Some gland cavities expanded, and mucus was accumulated in the cavities (Figure 2(a)). The cavities were lined purely by mucinous luminal cells, and a continuous layer of basal cells was under the mucinous luminal cells (Figure 2(b)). The mucinous luminal cells did not show obvious atypia, and the nucleus was located at the base of the cells. There was abundant intracellular and extracellular mucus (Figure 2(c)). Under the base of the epithelium were round basal cells, most of which were arranged in a single layer. The basal cell size was relatively uniform, and no mitosis or necrosis was found (Figure 2(d)). Basal cells in some areas were not obvious (Figure 2(e)). The outer stroma of the basal layer was interstitial cells, with lymphocytes and plasma cells infiltrating. There were abundant interstitial cells in some sections (Figure 2(f)). No pleural invasion was found, and no metastasis was found in the lymph nodes of group 11 (0/10).

#### 3.4.3. Immunohistochemical Results

**Figure 3:**
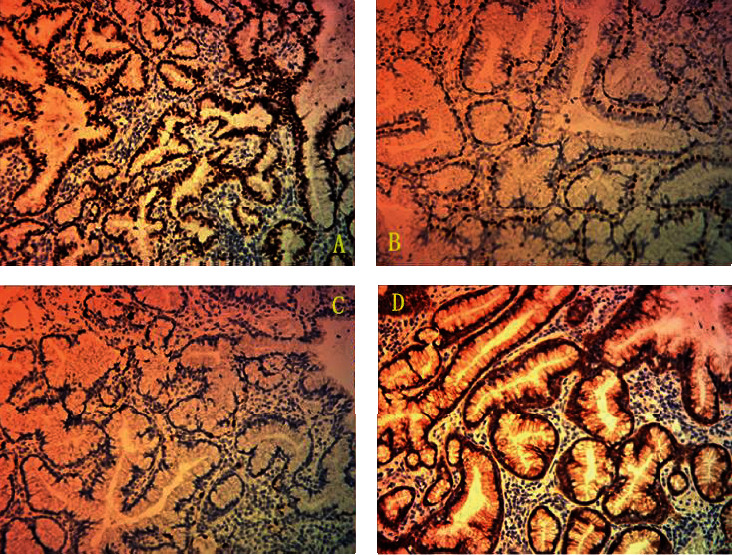
Immunohistochemistry. Both mucinous luminal cells and basal cells were positive for TTF1 (a) (200x). Basal cells were positive for p40 (b) (200x). Focal mucinous luminal cells were positive for NapsinA (c) (200x). Mucinous luminal cells were positive for EGFR (d) (200x).

P40, P63, CK5/6, and TTF-1 in continuous basal cells showed positivity (Figures 3(a) and 3(b)). SMA in interstitial cells showed positivity. CK7, Tyr1068 EGFR, and MUC1 in mucinous luminal cells all showed positivity, while NapsinA was focally positive (Figures 3(c) and 3(d)). Positive index of Ki-67 was about 2%.

#### 3.4.4. Histochemical Staining Results

The mucinous luminal cells and extracellular mucus were stained bluely by AB-PAS.

### 3.5. Follow Up Results

No tumor recurrence or metastasis was found in the patient's 2-year follow-up.

### 3.6. Final Diagnosis

The pathological diagnosis was BA (distal type) (5th edition WHO, ICD-O code: 8140/0).

## 4. Discussion

### 4.1. Definition and Overview

Ishikawa firstly described CMPT in 2002 [2]. Because of its structural characteristics, CMPT is easily misdiagnosed as IMA, especially in intraoperative frozen pathology. According to Shirsat et al.'s study [24], only 3 cases of 18 (16.7%) were diagnosed as BA/CMPT with frozen sections, and that was only 2 cases of 9 (22%) in another study [22]. Some scholars believed that CMPTs with obvious papillary structures were called classic CMPT, while CMPTs without typical papillary structures were called nonclassical CMPT [25]. Chang et al.'s study with 25 cases of CMPT found that the morphology and immunophenotype of both the classic and nonclassical CMPTs were similar to those of the bronchiole proximal to distal, especially the epithelial cells were similar to the mucosal epithelial cells of the end bronchioles [1]. Chang et al. firstly defined this type of tumor as BA, which was divided into proximal-type and distal-type. Some researchers believed that classic CMPT was a papillary subgroup of proximal BA, while nonclassical CMPT corresponded to distal BA [26]. According to these works, the World Health Organization applied BA to the 2021 edition of the classification of chest tumors.

### 4.2. Routine Pathological Diagnosis and Differential Diagnosis

#### 4.2.1. Histological and Immunohistochemical Diagnosis

The histological characteristic of BA is a double-layered cell structure formed by luminal cells and basal cells. Mucous cells, Clara's cells (apocrine-like cells), and ciliated cells often form adenoid or papillary structures in different proportions. There is a complete basal cell layer beneath the luminal cells [1, 20]. A large amount of mucus can be seen in the gland cavity and surrounding alveoli. Immunohistochemical staining showed that all luminal cells (ciliated cells, mucous cells, and Clara's cells) expressed CK7 but not CK20. Epithelial cells, except for mucous cells, expressed TTF-1, and the positive intensity was different. Reports showed that all luminal cells express NapsinA, and Clara's cells expressed CC10+. Basal cells express TTF1, p40, p63, and CK5/6. Ki-67 proliferation index of BA was very low [1, 20, 26]. The coloring of the mucus with AB-PAS staining of BA is deeper than the mucinous adenocarcinoma [27]. Only one case of BA lined purely by mucinous luminal cells has been reported in the English literature [23]. The histology, immunohistochemistry, and histochemical staining of our case were consistent with that of the literature.

#### 4.2.2. Differential Diagnosis

1. *AIS and IMA.* Distal type BA with glandular cavities covered only by alveolar cells or Clara's cells is very similar to in situ adenocarcinoma (AIS). BA lined purely by mucinous luminal cells is easily confused with IMA. These tumors can be identified by labeling basal cells with p40, p63, and CK5/6, and finding ciliated cells is also very useful. Literature reported that all BAs were small lesions (≤ 2 cm), while IMA varied in size [1].
2. *Micropapillary Adenocarcinoma.* BA with micropapillary clusters must be differentiated from micropapillary adenocarcinomas. The ciliated cells and basal cell layers in the capillary clusters of BA can be observed under a high-power microscope [1].
3. *Ciliated Adenocarcinoma.* Articles [28–30] reported rare ciliated adenocarcinomas. The distinguishing points (cytological atypia, naive cilia, and visible mitosis, but not double-layered cell structure) can be observed in ciliated adenocarcinomas. Some cases were initially diagnosed as ciliated adenocarcinoma and ultimately confirmed as proximal BA [31].
4. *Peribronchial Metaplasia (PBM).* PBM is a reactive metaplasia characterized by a two-layer cellular structure with ciliated and mucous cells. PBM is a small lesion (usually within 1 mm) involving multiple airways, often accompanied by pulmonary fibrosis and pneumonia. BRAF or EGFR mutation detection can help distinguish BA from PBM. Report [1] showed that IHC staining of BRAF in 9 cases of PBM was negative
5. *Mucinous Adenoma and Mucinous Systadenoma.* Both are benign tumors with an incomplete myoepithelial cell layer in the epithelial base rather than a continuous basal cell layer. Myoepithelial markers may be helpful in differential diagnosis with BA [23].
6. *Bronchial Papilloma.* Bronchial papilloma is usually located in the bronchus. A recent study has shown that the detection of AKT1 mutations can be helpful in differentiating from BA [28].

### 4.3. Intraoperative Frozen Pathological Diagnosis

The treatment plan of BA may be based on the results of intraoperative frozen pathology. However, due to factors such as unfamiliarity, time limit, and difficulty to make good slices with unfixed tissue, frozen pathological diagnosis of BA is challenging even for experienced pathologists. Seven cases of 9 (77.8%) reported by Chang et al. [1] were misdiagnosed as lung cancer, and 1 case was diagnosed as mucinous adenoma. Only 3 cases in 18 (16.7%) reported by Shirsat et al. [24] were diagnosed as BA, and 8 cases (44.5%) were misdiagnosed as IMA. Both basal cells and ciliated cells can help to eliminate IMA and mucoepidermoid carcinoma (MEC). BA is a single lesion (less than 2 cm) with clear boundaries [22]. Actually, for cases with challenge, pathologists can only make a descriptive diagnosis in intraoperative frozen pathology. It has to wait for routine pathological diagnosis.

### 4.4. Imaging Features of BA

CT plain scan shows that BA usually manifests as a single high-density ground-glass nodule, which was similar to early IMA. Central low-density thin-walled cavities can be seen in some BAs [6]. The enhanced CT scan shows ring enhancement in the solid part of BA, which is also similar to early IMA. In fact, the similarities can also be regarded as a feature of BA [5]. Sun et al. [19] were the first to distinguish BA from AIS and MIA with unenhanced thin-slice CT image texture analysis before surgery. Compared with AIS/MIA, false cavities are more common in BA [12]. IMA has the appearance of infiltrating and invading the pleura, and BA near the pleura usually only squeeze the pleura without traction or invasion [27].

### 4.5. Molecular Pathological Characteristics of BA

BA has certain unique molecular characteristics. The driver gene mutation frequency of BA is 70%-86%, and the mutation rate of the proximal type was higher than that of the distal type. The most common mutations include BRAF (38%), KRAS (24%), EGFR (19%), and HRAS (5%) [1, 8, 23]. Among the 66 cases reported in the English literatures, there were 16 BRAF V600E mutations, 2 BRAF G606R mutations, 7 EGFR mutations, 1 HRAS mutation, 5 KRAS mutations, 2 ALK rearrangements, and 1 AKT E17K mutation; the rest were 32 cases with unknown genetic changes or without genetic testing [20]. The specific mutant genes of BA were BRAF V600E, BRAF G606R, EGFR (del E746-T751/S752 V), EGFR (E709G), and KRAS (G12 V) [11]. Chang et al. [1] found 1 HRAS G13R hotspot and BRAF G464V mutation. EGFR and KRAS mutations occurred earlier in lung adenocarcinoma [2, 18]. P53 mutation might predict the malignant potential of BA [32]. Chang et al. [1] found high consistency between the immunohistochemical and NGS results of BRAF V600E.

### 4.6. Treatment and Prognosis of BA

BA is generally considered as a benign tumor. 66 cases in the English literatures that have been surgically resected were followed up for an average of 26 months (2-120 months), with no recurrence or metastasis. Wang et al. [20] found low expression (5% TPS) of PD-L1 (22C3) in BA. However, Miyai et al. [32] reported an extremely rare case of invasive CMPT with local atypical spindle interstitial cells and basal cells infiltrating the fibrous stroma in a reticular pattern. They believed that the basal cells underwent malignant transformation. So, postoperative follow-up is recommended for all BAs. Our case was followed up for 2 years and no recurrence or metastasis was found.
